# Herbal medicine: a survey of use in Nigerian presurgical patients booked for ambulatory anaesthesia

**DOI:** 10.1186/1472-6882-12-130

**Published:** 2012-08-20

**Authors:** Tonia C Onyeka, Humphrey A Ezike, Ogochukwu M Nwoke, Emeka A Onyia, Elias C Onuorah, Samson U Anya, Timothy E Nnacheta

**Affiliations:** 1 Department of Anaesthesia, University of Nigeria Teaching Hospital, Ituku-Ozalla, Enugu, PMB 01129, Nigeria

**Keywords:** Herbal medicine use, Ambulatory anaesthesia, Day case surgery

## Abstract

**Background:**

Utilization of herbal medicines in the preoperative period by Nigerian patients booked for day case surgery has not been explored.

**Methods:**

Cross-sectional survey of 60 patients presenting for day-case surgery at a tertiary healthcare institution over a 3-week period in August 2011 was conducted. Using a structured questionnaire, inquiries were made concerning use of herbal medicines in the immediate preoperative period. Socio-demographic characteristics, information on use of concurrent medical prescriptions, types of herbs used, reasons for use, perceived side effects and perceived efficacy were obtained. Data were evaluated using descriptive statistics and Chi-square.

**Results:**

Fifty-two (86.7%) were American Society of Anesthesiologists (ASA) class 1 while 8 (13%) were ASA 2. Most patients (86.7%) had their procedures done under local infiltration with monitored anaesthesia care (MAC), while 5.0% and 8.3% had their procedures done under regional and general anaesthesia, respectively. About 48.3% of respondents were on concurrent medical prescriptions while 51.7% were not. Forty percent (40%) of patients admitted to use of herbal medicine, all by the oral route, in the immediate perioperative period; 87.5% did not inform their doctor of their herbal use. Types of herbs used included ‘dogonyaro’, ‘agbo’, ‘nchanwu’, and Tahitian noni. Treatment of malaria was commonest reason for use in 29.2% of patients, while cough and concurrent surgical condition were reasons given by 12.5% of patients, respectively. Seventy-nine percent (79.2%) of patients considered their herbal medications effective. Perceived side effects of herbal medication (16.6%) included fever, waist pain and intoxication. There were no variations in use between ASA 1 and ASA 2 patients and none between respondents on conventional medication against those that were not. Variables such as age less than 35 years, female gender, being married and being an urban dweller did not show any significant difference in use.

**Conclusion:**

This survey revealed many patients were on one or more herbal preparations in the immediate preoperative period. In consideration of possible untoward drug interactions between conventional medication, herbal preparations and anaesthesia, doctors (especially anaesthetists) should routinely assess all patients booked to be anaesthetized, especially those for day case surgery. The authors recommend surveys with larger respondent numbers to determine prevalence of use and possible interactions between indigenous Nigerian herbs and anaesthesia.

## Background

Use of herbal remedies is not a strange phenomenon in the African society. For instance, 80% of Africans [1] and about 27 million South Africans (54%) have been identified as herbal remedy users [2]. General use of herbal medicine amongst Nigerians is also well documented. Ezeome et al. [3] determined that use of herbs and other forms of complementary and alternative medicine (CAM) was common among cancer patients in Nigeria. Other studies have revealed its use in children [4], asthma patients [5], hypertensive patients [6], pregnant women [7,8], as well as medical inpatients [9] and outpatients [9,10]. However, no studies exist presently to determine the use of herbal medicine among the presurgical patient population presenting for ambulatory or day case anaesthesia in Nigeria. Ambulatory anaesthesia is used for surgical procedures where the patient is not required to stay overnight in the hospital. Local, regional or general anaesthesia as well as monitored anaesthesia care (MAC) are the forms in which ambulatory anaesthesia can be conducted.

There are several reasons given as to why people contemplate herbal medicine use. While some consider them to be natural and therefore safe [11,12], others put economic reasons into consideration: high cost of healthcare and poor access to conventional medications. Protracted health issues [13], religion and traditional or cultural beliefs [14] have been known to play a role as well as dissatisfaction with efficacy of conventional medications [15] and the perception that herbal remedies are much more efficacious than the former [16].

The pharmacologic properties of most herbs remain uncertain as a result of a lack of extensive research in this area. Further compounding this issue is a lack of standardization and incomplete regulation of herbal remedies, contamination with pesticides [13], microorganisms or heavy metals, adulteration, improper labelling and incorrect dosing [16,17]. It has been established that there is increased risk of adverse reactions from herbal medicine interaction with medical prescription or anaesthesia. These include effects on coagulation, leading to postoperative bleeding [18], sedation as a result of the presence of conventional medicine additives to the herbal preparation such as diazepam [19], and nephrotoxicity from the presence of heavy metals [16]. In addition, prolonged or inadequate anaesthesia has been known to occur in patients on herbal remedies in the preoperative period [20]. In this survey, we set out to establish use and determine nature of herbal use among patients to be anaesthetized for day case surgery in our institution. The University of Nigeria Teaching Hospital, which is a 500-bedded tertiary hospital is located at Ituku-Ozalla, Enugu, South-East Nigeria, the latter being a region occupied predominantly by the Igbo ethnic group.

## Methods

This cross-sectional survey was undertaken as a pilot study for a larger national multicenter survey. Sixty (60) adult patients for ambulatory anaesthesia were enrolled over a 3-week period in August 2011 for the survey. The questionnaire was adapted from a similar study [21] to which more questions were added. The modified questionnaire ( Additional file 1) was tested on fifteen (15) pre-surgical patients to ensure it was easy to understand. Names of locally available herbs and herbal remedies were incorporated into the questionnaire prior to its administration. Provisions were made in the questionnaire for unknown herbs and herbs identified by their vernacular names. Following approval by the University of Nigeria Teaching Hospital Research and Ethics Committee, literate respondents filled the questionnaires themselves while illiterate ones had the questionnaire administered to them by medical residents as they underwent pre-anaesthetic evaluation. Respondents were informed of the survey and had the option to accept or decline involvement. The response rate was 80%. Although the low numbers of patients were booked for ambulatory anaesthesia within the survey period, findings from this survey would serve as a guide to much larger studies, especially as no previous data on herbal medicine intake in the Nigerian presurgical patient exists.

### Participants

Respondents enrolled into this survey were American Society of Anesthesiologists (ASA) class 1 and 2 adult patients presenting consecutively for day case surgery. Paediatric patients (i.e. under 18 years of age), ASA 3 and 4 patients and patients for emergency surgical procedures were excluded from the survey.

### Data collection and analysis

In addition to socio-demographic and herbal use data, other variables included clinical diagnosis, ASA class, operation, type of anaesthesia, presence or absence of concomitant medical conditions as well as concurrent medical prescriptions. Others were types and number of herbs used, reasons for use, perceived side effects and perceived efficacy of the herbal medicines. Analysis of data in the form of frequencies was conducted using SPSS 19 statistical software. Chi-square was used to characterize nominal variables such as age, sex, marital status, etc.

## Results

### Respondent characteristics

A total of 60 respondents, all of the Igbo ethnic group, presenting with a wide array of surgical conditions and with a female predominance (55%), were interviewed. Of these, 87% were American Society of Anesthesiologists (ASA) class 1 and 13% were ASA class 2. Respondents found to take herbal preparations had an average age of 21.5 years (Table 1). While 45% of respondents possessed tertiary education, 66.7% were rural dwellers. Most respondents (86.7%) had their procedures done under local infiltration with monitored anaesthesia care (MAC), while 5.0% and 8.3% had their procedures done under regional and general anaesthesia, respectively. Regarding concomitant medical conditions, 46.7% were known hypertensive while 13.3% were either diabetic or had Koch’s disease (Table 2). About 48.3% of respondents were on various concurrent medical prescriptions while 51.7% were not. Of these medications, 8 were identified while a good number (41.4%) of them were unknown.

**Table 1:** Socio-demographic characteristics

**Variable**	**Frequency (n=60)**	**Percentage**
**Age**		
18–25	17	28.3
26–35	12	20.0
36–45	8	13.3
46–55	6	10.0
56–65	6	10.0
>65	11	18.3
**Sex**		
Male	27	45.0
Female	33	55.0
**Marital Status:**		
Single	26	43.3
Married	33	55.0
Widowed	1	1.7
**Educational level**		
None	7	11.7
Primary	8	13.3
Secondary	18	30.0
Tertiary	27	45.0
**Location**		
Urban	40	33.3
Rural	20	66.7

**Table 2:** Medical Co-morbidities of Respondents

**Variable**	**Frequency (n=15)**	**Percentage**
**(n=15)**		
Hypertension	7	46.7
Peptic Ulcer Disease	1	6.7
Diabetes Mellitus	2	13.3
Asthma	1	6.7
Tuberculosis	2	13.3
Migraine	1	6.7
Other	1	6.7

### Herbal use

Forty percent (40%) of respondents reported the use of herbal medicine in the immediate perioperative period, of which 75% used between 1 and 2 herb types. All respondents who took herbal medications did so by the oral route, most (62.5%) obtaining them through family members and friends and 87.5% did not inform their doctor of their herbal use. Types of herbs mentioned included aloe vera, ginseng, ‘onugbu’, ‘dogonyaro’, ‘agbo’, ‘ugu’, ‘utazi’, ‘nchanwu’, garlic, ginger, Tahitian noni (the propriety name for noni juice, an extract from the Indian mulberry tree, *Morinda citirifolia*) as well as ‘unknown’ and alcohol-containing herbal preparations. Overall, fourteen different herbs were found to be used by this population, as reported by respondents. The most common reason for use in 29.2% of the respondents was the treatment of malaria, while cough and the concurrent surgical condition were reasons given by 12.5% of respondents, respectively (Table 3). Seventy-nine percent (79.2%) of respondents considered their herbal medications effective. Side effects of the herbal preparation as perceived by 16.6% of respondents were fever, waist pain and intoxication (light-headedness). There were no variations in herbal remedy use between ASA 1 and ASA 2 respondents (p=0.24) and none between respondents on conventional medication against those that were not (p=0.10). Similarly, variables such as age less than 35 years (p=0.48), female gender (p=0.18), being married (p=0.46) and being an urban dweller (p=0.39) did not seem to show any significant difference in use.

**Table 3:** Reasons for herb use

**Variable**	**Frequency (n=25)**	**Percentage**
Malaria	7	29.2
Cough	3	12.5
Concurrent surgical condition	3	12.5
Malaise	2	8.3
Abdominal discomfort	2	8.3
‘Stomach cleansing’	2	8.3
‘Anti-poison’	2	8.3
Typhoid	1	4.2
Amenorrhea	1	4.2
General well being	1	4.2
Pain relief	1	4.2

## Discussion

This exploratory survey was carried out to establish the use of herbal medications by patients presenting for ambulatory or day case anaesthesia in our institution. The increasing global popularity of day case surgery and consequently ambulatory anaesthesia have been attributed to numerous advantages such as reduction in hospital costs (e.g. hospitalization and bed space) [22], shorter waiting lists, patient’s ease and less disruption to daily life activities, quick recovery and reduced postoperative demands like analgesics [23]. The results of this survey show that herbal medicine use occurs among patients for ambulatory anaesthesia. A number of studies conducted in Europe, Australia and the United States [12,13,20] have shown use of popular herbs like gingko, St. John’s Wort, ginseng, ginger and garlic. Findings from the present survey revealed use of common herbal medicines such as garlic, ginger and Tahitian noni, as well as lesser known indigenous herbs like ‘dogonyaro’, ‘agbo’, ‘nchanwu’, ‘ugu’, ‘onugbu’ and ‘utazi’. This is the first survey of its kind as far as we know as no previous work has examined for use of indigenous Nigerian herbs as well as foreign herbs in the preanaesthetic period and among day case patients.

Of the 25 respondents who reported to use herbs, 75% used a maximum of two herbs and an equally high percentage (79.2%) of them perceived the herbal preparations to be effective, despite their admittance of side-effects (66.7%). Majority of respondents (87.5%) did not inform their doctors of herbal medicine use unlike similar studies where rates of patient disclosure is high [24]. As previous studies have noted, non-disclosure increases the potential for untoward drug-herb-anaesthetic reactions in the perioperative period [21]. Although this survey did not probe into reasons for non-disclosure, belief that herbs are more potent than conventional medicines, that doctors lack knowledge of herbs, ignorance about the potentials for adverse reactions, fear of admitting use as well as not being asked specifically about its use [21] are some of the reasons adduced. The obligation therefore lies on healthcare practitioners to elicit history of herbal use during preoperative assessment.

Tahitian noni is used by many to treat inflammatory conditions such as abscesses and for general well-being as indicated by patients who admitted to its use in this survey. There are unsubstantiated claims of its ability to lower blood sugar levels in diabetics. However, animal studies conducted using this extract has shown it to have both anti-nociceptive and anti-inflammatory effects and the potential to cause hyperkalemia [25] and acute hepatitis with regular use [19] has been documented.

‘Dogonyaro’, a name derived from the Hausa language of the Hausa-Fulani tribe of Northern Nigeria, is the name given to the Neem tree (*Azadirachta indica*) found more commonly in these parts. Widely used as chewing stick among the populace, unsubstantiated uses include the treatment of stomach ulcers and for birth control. While noted to have analgesic and anti-inflammatory activities in animal models [26], it has been shown to have potential hepatotoxic [27], nephrotoxic [28] and hypoglycaemic [29] properties in animal models.

‘Nchanwu’, so-named by the Igbos of South-East Nigeria, is the holy basil plant (*Ocimum viride*). Commonly called ‘scent leave’ by locals and ‘efinrin wewe’ by the Yorubas of South-West Nigeria, it is also a popular food spice. It is claimed to cure asthma, earaches and skin diseases [30] and was used by some patients in this survey for treatment of common cold and catarrh. It has demonstrated apoptotic and cytotoxic activity towards human colorectal adenocarcinoma cells [31]. Apart from exhibiting hypoglycaemic properties [32], studies suggest it may have COX-2 inhibitor effect [33].

‘Agbo’ is an herbal concoction used by many Nigerians as well as the Yoruba tribe of South-West Nigeria from where it derives its name. Made up of several constituents such as alcohol/palm wine, water, lime, tea leaf, various tree barks & roots in varying proportions, ‘agbo’ is claimed to cure fevers, back ache, cause an increase in libido as well as cure virtually all manner of maladies. Some patients in this study admitted to use of this concoction for relief of their concurrent surgical condition, abdominal discomfort, ‘stomach cleansing’ and as ‘anti-poison’ amongst other uses. Chronic consumption of ‘agbo’ preparations, particularly those with alcohol or palm wine base, has the potential to cause alcoholic hepatitis and cirrhosis.

‘Utazi’ (*Gongronema ratifolia*), ‘Ugu’ ( *Telfaria occidentalis*) or fluted pumpkin, and ‘Onugbu’ ( *Vernonia amygdalina*) are green leafy vegetables commonly used by the Igbos of South-East Nigeria for food preparation as well as for medicinal purposes. The leaves of the fluted pumpkin, purported by traditional herbalists to increase sperm count and improve reproductivity, has been noted to contain nitrates and oxalates which have the potential to cause methhaemoglobinaemia and kidney stones respectively with high intakes [34].

## Conclusion

Use of one or more herbal remedies in oral form, in the immediate preoperative period by patients booked for day case surgery was observed. Even though use was not dependent on gender, age, location or educational level attained, these results cannot be considered to be representative of all patients coming for day case surgery because of the sample size used. Many herbal preparations alluded to in this survey have potentially deleterious effects on patient well-being in the immediate postoperative period [35]. The authors recommend surveys with larger respondent numbers to determine prevalence of use and possible interactions between indigenous Nigerian herbs and anaesthesia. Majority did not inform their doctors of their concomitant use of herbal remedies and conventional medication. Doctors, especially anaesthetists, should be aware of the potential for interactions between conventional medications, herbal medicines and anaesthesia and imbibe the culture of routine evaluation for herbal use – herbal pharmacovigilance [36] – by instituting a herbal preoperative policy in their institutions and include herbal documentation in the anaesthetic charts, especially those for day case surgery. It would also be good practice to ask patients to discontinue all herbal medications, two weeks prior to the scheduled surgical procedure.

## Abbreviations

ASA, American Society of Anesthesiologists; CAM, Complementary and Alternative Medicine; COX 2, Cyclo-Oxygenase 2 Inhibitor; IUCD, Intrauterine Contraceptive Device; MAC, Monitored Anaesthesia Care.

## Competing interests

The authors declare that they have no competing interests.

## Authors’ contribution

OTC conceived the study, designed the questionnaire, conducted the analysis and was involved in the writing of the manuscript. EHA, NOM, OEC, AS, OCE and NTE were involved in the distribution of the questionnaire, collation of data and the writing of the manuscript. All authors read and approved the final manuscript.

## Pre-publication history

The pre-publication history for this paper can be accessed here:

http://www.biomedcentral.com/1472-6882/12/130/prepub

## Supplementary Material

Additional file 1 **Appendix.** Questionnaire. Click here for file
