# Supplementary material for: Herbal medicine: a survey of use in Nigerian presurgical patients booked for ambulatory anaesthesia
Source: BMC Complement Altern Med. 2012 Aug 20;12:130. doi: 10.1186/1472-6882-12-130 (PMC3439280; doi:10.1186/1472-6882-12-130)
Supplement: Additional file 1 — Appendix. Questionnaire. [file 1472-6882-12-130-S1.doc]

*APPENDIX* *1*

*QUESTIONNAIRE*

1. AGE: □ 18 – 20 □ 21 – 25 □ 26 – 30 □ 31 – 35 □ 36 – 40 □41 – 45 □46 – 50 □51 – 55 □56 – 60 □61 - 65 □ >65
2. SEX: □ Male □ Female
3. MARITAL STATUS: □ Single □ Married □ Widowed □ Divorced
4. OCCUPATION: …………………………………………………………………………….
5. RELIGION: ………………………………………………………………………………...
6. EDUCATIONAL LEVEL: □ None □ Primary □ Secondary □ Tertiary
7. LOCATION: □ Rural □Urban
8. CLINICAL DIAGNOSIS: ………………………………………………………………….
9. OPERATION: ……………………………………………………………………………...
10. CONCOMITTANT MEDICAL CONDITION: …………………………………………...
11. ASA: □ 1 □ 2
12. TYPE OF ANAESTHESIA: □ Local MAC □ Regional □ GA
13. CURRENT USE OF MEDICAL PRESCRIPTION(S)?: □ Yes □ No
14. MEDICAL PRESCRIPTION(S) …………………………………………………………...
15. ROUTINE USE OF HERBAL PREPARATIONS?: □ Yes □ No
16. NO. OF HERBAL PREPARATIONS USED: □ 1 - 2 □ 3 - 4 □ >4
17. HERBAL MED TYPE:

| 1. Aloe vera | 1. Garlic | 1. Ginger | 1. Ginseng | 1. ‘Tahitian Noni’ | 1. ‘Dogonyaro’ | 1. Paw-paw leaves | 1. ‘Onugbu’ |
| --- | --- | --- | --- | --- | --- | --- | --- |
| 1. ‘Nchanwu’ | 1. Alcohol-containing prep | 1. ‘Ugu’ | ‘Agbo’ | 1. ‘Utazi’ | 1. Unknown | 1. Others |  |
|  |  |  |  |  |  |  |  |
|  |  |  |  |  |  |  |  |

1. ROUTE OF ADMINISTRATION: 1. ……… 2. ………3. ………. 4……………….
2. REASONS FOR USE: ………………………………………………………………
3. IS IT EFFECTIVE?: □Yes □ No □ Not sure

21. SOURCE OF HERBAL PREP: □ Herbal practitioner □ Book □ Media □ Internet □ Family/friends

22. IS YOUR DOCTOR AWARE THAT YOU ARE TAKING IT?: □ Yes □ No

23. DOES THE HERBAL PREP HAVE ANY SIDE EFFECTS?: □ Yes □ No □ Not sure

24. IF YES, WHAT ARE THEY? ……………………………………………………………...
